# Mental imagery during daily life: Psychometric evaluation of the Spontaneous Use of Imagery Scale (SUIS)

**DOI:** 10.5334/pb.ag

**Published:** 2014-01-20

**Authors:** Sabine Nelis, Emily A. Holmes, James W. Griffith, Filip Raes

**Affiliations:** 1 Faculty of Psychology and Educational Sciences, University of Leuven, BE; 2 MRC Cognition and Brain Sciences Unit, Cambridge, UK; 3 Northwestern University and University of Leuven

**Keywords:** mental imagery, validation, factor analysis, questionnaire

## Abstract

The Spontaneous Use of Imagery Scale (SUIS) is used to measure the tendency to use visual mental imagery in daily life. Its psychometric properties were evaluated in three independent samples (total *N* = 1297). We evaluated the internal consistency and test-retest reliability of the questionnaire. We also examined the structure of the items using exploratory and confirmatory factor analysis. Moreover, correlations with other imagery questionnaires provided evidence about convergent validity. The SUIS had acceptable reliability and convergent validity. Exploratory and confirmatory factor analysis revealed that a unidimensional structure fit the data, suggesting that the SUIS indeed measures a general use of mental imagery in daily life. Future research can further investigate and improve the psychometric properties of the SUIS. Moreover, the SUIS could be useful to determine how imagery relates to e.g. psychopathology.

Mental imagery has received considerable interest in the domain of cognitive neuroscience, experimental psychopathology and clinical research (e.g., intrusive images, suicidal images, use of mental imagery in therapy; Holmes & Mathews, 2010). Kosslyn, Ganis and Thompson (2001) referred to mental imagery as the experience of ‘seeing with the mind’s eye’, ‘hearing with the mind’s ear’, and so on. Visual mental imagery was described by Kosslyn (1987) as “For example, visual imagery is usually identified as producing “the experience of seeing in the absence of the appropriate sensory input” or the like. Having a visual mental image produces the conscious experience of “seeing,” but with the “mind’s eye” rather than with real ones” (p. 149). The Spontaneous Use of Imagery Scale (SUIS; Reisberg, Pearson, & Kosslyn, 2003) is a 12-item self-report scale that was developed to measure self-reported spontaneous use of mental imagery in daily life (initially referred to as *unpublished observations* of S.M. Kosslyn, J. Shepard, W.L. Thompson, and C.F. Chabris; e.g., in Mast, Ganis, Christie, & Kosslyn, 2003). It is sometimes described as a *trait measure of mental imagery use* (e.g., McCarthy-Jones, Knowles, & Rowse, 2012, see also Pearson, Deeprose, Wallace-Hadrill, Burnett Heyes, & Holmes, 2013). The SUIS focuses only on the use of visual imagery; information on other modalities (e.g., auditory imagery) is not measured by the SUIS. On the SUIS, people indicate how often a certain description is appropriate for them, from *always completely appropriate* to *never appropriate*. Each description concerns a daily life situation in which images are involved, images come into your mind, or in which images might be useful. In this sense, the SUIS focuses on the frequency and likelihood of mental imagery during daily life. In contrast, other measures of imagery skills (e.g., Vividness of Visual Imagery Questionnaire (VVIQ), Marks, 1973; and Questionnaire upon Mental Imagery (QMI), Sheehan, 1967) focus on the quality and vividness of mental images. Reisberg et al. (2003) first reported the psychometric properties of the SUIS, including internal consistency and the relationship with another imagery questionnaire. The purpose of Reisberg et al.’s (2003) study was to explore how researchers’ experiences with mental imagery influence their theoretical views on mental imagery; participants were psychologists, philosophers, and neuroscientists. Good reliability was reported based on high corrected item-total correlations >= 0.98. Participants experiencing a high level of image vividness had a higher SUIS score than ‘low-vividness’ imagers. McCarthy-Jones, Knowles, and Rowse (2012) reported good internal consistency of the SUIS measured by Cronbach’s alpha (*α* = .83). To our knowledge, information on the factor structure of the SUIS has not been published.

Since 2003, the SUIS has been used in various studies. Holmes et al. (2011), for example, used it to investigate imagery use in patients with bipolar disorder. Price (2009) used it to examine imagery use in people experiencing ‘synaesthetic’ spatial forms. Across studies, researchers have used it as a control measure to check whether participants’ baseline use of imagery differs between groups in studies investigating mental imagery (e.g., Davies, Malik, Pictet, Blackwell, & Holmes, 2012; Holmes, Lang, Moulds, & Steele, 2008; Mast et al., 2003).

The present study had two objectives. First, the original, English version of the SUIS was translated into Dutch. Second, we examined the psychometric properties of the Dutch version. In three separate samples, we used factor analyses to determine the underlying structure of the data. We also examined test-retest reliability and internal consistency of the SUIS scores, as well as convergent validity using concurrent measures of imagery vividness.

## Method

### Participants

Three samples were included: Sample 1 consisted of 495 first-year psychology students from the University of Leuven who participated in return for course credit. Four participants had omissions for all SUIS items and/or demographic info and were excluded, resulting in a sample of 491 participants. In Sample 2, we recruited a heterogeneous, community-based sample via e-mail. They participated without compensation, financial or otherwise. Forty-six participants did not complete the SUIS and were consequently not included in our analyses. We also excluded five participants with missing data for age and/or gender. The remaining sample consisted of 373 respondents. In terms of education, 50% of the respondents had a university degree (bachelor’s or master’s), 22% had a professional bachelor degree at a university college, 4% followed a specific professional education after secondary school, 24% completed primary or secondary school. Sample 3 consisted of 437 first-year psychology students from the University of Leuven who participated in return for course credit. Four participants were excluded because they had omissions for all SUIS items, completed less than 80% of the SUIS items, or had missing data on age. This left a sample of 433 participants. Information on age and gender for each sample is shown in Table 1.

**Table 1:** Descriptive statistics

		Raw data						POMP (percentage of maximum possible)		

	**Age** ***M* (*SD*)** ***Min-Max***	**Gender** **(*% female*)**		***α***	***M* (*SD*)**	***Min***	***Max***	***M* (*SD*)**	***Min***	***Max***

**Sample 1** ***N* = 491**	18.6 (1.8) 17–36	82	**SUIS**	.76	38.7 (7.8)	15	57	56 (16)	6	94

**Sample 2** ***N* = 373**	34.9 (15.5) 16–76	68	**SUIS**	.72	37.9 (7.4)	16	56	54 (15)	8	92

			**VVIQ**	.89	36.4 (11.4)	16	80	32 (18)	0	100

			**QMI visual**	.85	13.0 (5.8)	5	35	27 (19)	0	100

**Sample 3** ***N* = 433**	18.4 (1.8) 17–44	81	**SUIS**	.72	40.1 (7.5)	19	60	58 (16)	15	100

*Note. α* is based on participants who completed the whole questionnaire. *Mean, SD, MIN* and *MAX*, age and *gender* are based on participants who completed at least 80% of the questionnaire (= reported *N*)

### Materials

**Vividness of Visual Imagery Questionnaire (VVIQ; Marks, 1973).** The Vividness of Visual Imagery Questionnaire measures mental imagery ability in terms of vividness and consists of four scenes (relative/friend, rising sun, a shop, a landscape). Each scene is divided into four specific aspects which have to be visualized (e.g., the color and shape of the trees). Participants rated all items on image vividness using a 5-point scale (‘*perfectly clear and vivid as normal vision when I really look at something’* to ‘*no image at all, I only “know” that I am “thinking” about something’*). Total scores range from 16 to 80 with higher scores indicating weaker imagery ability. There were no instructions about whether the participants should keep their eyes closed or open while rating the vividness. Marks (1973) reported good reliability; Cronbach’s alpha in the current study was 0.89.

**Questionnaire upon Mental Imagery (QMI; Sheehan, 1967).** The Questionnaire upon Mental Imagery is a 35-item measure of mental imagery ability for seven sensory modalities (visual, auditory, cutaneous, kinesthetic, gustatory, olfactory, and organic). Participants have to indicate how clearly they can imagine a series of situations (e.g., image of a friend, taste of jam) on a 7-point vividness rating scale (*‘Perfectly clear and as vivid as the actual experience’* to *‘I think about it but I cannot imagine it’*). The present study focuses on the 5-item visual subscale, with a total score ranging from 5 to 35. Higher total scores indicate weaker imagery ability. Cronbach’s alpha in the current study was 0.85.

**Spontaneous Use of Imagery Scale (SUIS; Reisberg, et al., 2003).** The SUIS is a 12-item questionnaire designed to measure spontaneous use of imagery during daily life. Participants use a 5-point scale to rate the degree to which each item is appropriate for them (from *never appropriate* to *always completely appropriate*). A sample item is: “When I think about visiting a relative, I almost always have a clear mental picture of him or her”. A total score can be calculated by summing the 12 item scores, resulting in a total score ranging from 12 to 60 with higher scores indicating more use of mental imagery in everyday life. In the present study, we developed a Dutch version of the SUIS. After translating the items and instructions from English, the Dutch items were back-translated to English by a native Dutch speaker with a Ph.D. degree in English Literature and expertise in the translation and revision of academic documents, including questionnaires. These back-translations were reviewed by Dr. W.L. Thompson, a collaborator of Dr. Stephen Kosslyn, one of the SUIS authors. After the review by Dr. Thompson, we addressed three minor comments, leading to the final Dutch version of the SUIS (Appendix).

### Procedure

For Sample 1 and Sample 3, the procedure was as follows: at the beginning of the academic year, participants were invited by email to attend a paper-and-pencil mass-testing session. They received a booklet containing a consent form, a demographics form, the SUIS, and other questionnaires not used in this study. We used *snowball sampling* via email to obtain participants for Sample 2. Members of our lab invited friends and family to participate using invitation e-mails that contained the address of an online questionnaire tool, as well as a request to further distribute the invitation and online survey. On the corresponding website, participants completed demographic information, SUIS, VVIQ, QMI, and other questionnaires not used in this study.

### Analyses

Participants who completed at least 80% of the SUIS items were included in factor analyses. We used both exploratory factor analysis as well as confirmatory factor analysis to investigate the structure of the SUIS. First, exploratory factor analysis was used in Sample 1. Next, we sought to confirm this structure in Samples 2 and 3.

For the exploratory factor analysis, we used parallel analysis and Velicer’s minimum average partial (MAP) to determine the number of factors to retain (e.g., Hayton, Allen, & Scarpello, 2004; Horn, 1965; Velicer, 1976). O’Connor’s (2000) SPSS syntax was used for both analyses, which were conducted in SPSS Statistics 20. For parallel analysis, the distribution of the eigenvalues was created via 1000 iterations. The 95^th^ percentile criterion was used as the comparison baseline given recommendations in the literature (e.g., Glorfeld, 1995). We used principal components analysis versus factor analyses in the context of the parallel analysis because in some cases the latter method leads to over-extraction. Eigenvalues of the real data were based on polychoric correlations extracted from Mplus, which were also used for Velicer’s MAP. After parallel analysis and Velicer’s MAP, we conducted an exploratory factor analysis in Mplus, version 6.12. Parameters estimation was based on *WLSMV (“Weighted least square parameter estimates using a diagonal weight matrix with standard errors and mean- and variance-adjusted chi-square test statistic that use a full weight matrix”*, Muthén & Muthén, 1998–2010, p. 533) and a GEOMIN oblique factor rotation was used. In order to allocate items to factors, a threshold of 0.30 for factor loadings was used as guideline.

We used confirmatory factor analysis to determine whether the SUIS measures one single construct, or whether a different structure would provide a better fit. The fit of the models was assessed using the following indices: the comparative fit index (CFI; Bentler, 1990; Hu & Bentler, 1999), the Tucker-Lewis Index (TLI; McDonald & Marsh, 1990), the root mean square error of approximation (RMSEA; Browne & Cudeck, 1993), and the Chi-square test for model fit (χ^2^). The Chi-square was calculated using mean WLSMV (Muthén & Muthén, 1998–2010), therefore the χ^2^ value and degrees of freedom cannot be used in the usual ways in the interpretation of this fit index. A good model fit was indicated with *CFI* and *TLI* >= 0.95, *RMSEA* =< 0.06 (Hu & Bentler, 1999). Although we used cutoffs as guides, we also relied on our judgment to determine how well a model fit. Therefore we did not reject models if the indices slightly violated the acceptable cutoffs (Marsh, Hau, & Wen, 2004). We also calculated corrected item-total correlations, an intraclass correlation coefficient with a follow-up measure of the SUIS (5 months), and Cronbach’s alpha to further interpret the data in the context of classical test theory.

Besides the SUIS, respondents in *Sample 2* completed two other imagery questionnaires translated from English: the VVIQ and the visual subscale of the QMI. We used these questionnaires as criteria to investigate the validity of the SUIS. Pearson correlations were calculated. We only focused on the visual subscale of the QMI given that the SUIS is mainly visual. It is important to mention that the visual subscale of the QMI is strongly comparable to five items of the VVIQ.

## Results

**Descriptive statistics.** Means, standard deviations, and range of the observed scores for the imagery questionnaires (SUIS, VVIQ, QMI) are reported in Table 1, subdivided by sample. To aid in interpretation, we also present descriptive statistics in terms of percent-of-maximum possible (POMP) scores (Cohen, Cohen, Aiken, & West, 1999). In inspecting frequency distributions for items of the SUIS, we observed that only 30 persons (of the 1297 across the three samples) indicated to having *never experienced* visual imagery in more than half of the 12 situations. In all three samples, women scored higher than men on the total SUIS score (Sample 1: *M (SD)*_men_ = 37.0 (7.8), *M (SD)*_women_ = 39.1(7.8), *F*(1, 489) = 5.3, *p* = .02; Sample 2: *M (SD)*_men_ = 36.2 (7.8), *M (SD)*_women_ = 38.6 (7.1), *F*(1, 371) = 9.2, *p* = .003; Sample 3: *M (SD)*_men_ = 36.7 (7.8), *M (SD)*_women_ = 40.9 (7.2), *F*(1, 431) = 21.7, *p* <.001). Participants’ age was not significantly correlated with SUIS total score in Sample 2, *r*(373) = -.05, *p* = .37. Correlations with age were not calculated for the two datasets with students given the limited age variability.

**Exploratory factor analysis (Sample 1).** Parallel analysis using 1000 iterations suggested the extraction of two components: the first two observed eigenvalues were 3.92 and 1.30 compared with 1.33 and 1.24 for the 95^th^ percentiles of the randomly-generated data. All other eigenvalues were less than 1. For the second component, the observed eigenvalue was close to the 95^th^ percentile of the random data. In case of similar values, O’Connor (2000) recommends repeating the parallel analysis with a larger number of iterations. Repetition of the analysis with 10,000 iterations also suggested two components. Velicer’s MAP suggested extracting one component with a smallest average squared partial correlation of .02.

Taken together, Velicer’s MAP suggested one component. Parallel analysis suggested two components based on strict cutoffs. Thus, we investigated one- and two-factor models. Geomin factor loadings are depicted in Table 2. The two-factor model was as having one factor consisting of items three, four and eight; the second factor contained all other items except item one.

**Table 2:** Model fit indices and factor loadings for exploratory and confirmatory factor analysis

	Sample 1 EFA One-factor model	Sample 1 EFA Two-factor model	Sample 2 CFA One-factor model	Sample 2 CFA Two-factor model	Sample 3 CFA One-factor model	Sample 3 CFA Two-factor model

**CFI**			0.93	0.94	0.91	0.92

**TLI**			0.92	0.93	0.89	0.90

**RMSEA**			0.06	0.06	0.07	0.07

**90% CI for RMSEA**			0.04–0.07	0.04–0.07	0.06–0.08	0.06–0.09

**χ^2^**			115.50 *df* = 54 *p* < .001	95.87 *df* = 43 *p* < .001	174.19 *df* = 54 *p* < .001	145.39 *df* = 43 *p* < .001

**Factor loadings**						

	**F1**	**F1**	**F2**	**F1**	**F1**	**F2**	**F1**	**F1**	**F2**

Item 1	**0.30**	0.12	0.25	0.26	-	-	0.18	-	-

Item 2	**0.51**	**0.71**	-0.24	**0.47**	**0.47**		**0.41**	**0.41**	

Item 3	**0.51**	0.20	**0.43**	**0.49**		**0.58**	**0.51**		**0.61**

Item 4	**0.33**	0.08	**0.35**	**0.35**		**0.38**	**0.40**		**0.47**

Item 5	**0.55**	**0.50**	0.09	**0.49**	**0.49**		**0.62**	**0.63**	

Item 6	**0.41**	**0.54**	-0.15	0.19	0.19		0.28	0.29	

Item 7	**0.42**	**0.32**	0.16	**0.39**	**0.39**		**0.49**	**0.49**	

Item 8	**0.56**	0.00	**0.84**	**0.55**		**0.65**	**0.45**		**0.55**

Item 9	**0.67**	**0.52**	0.23	**0.55**	**0.56**		**0.65**	**0.66**	

Item 10	**0.74**	**0.73**	0.04	**0.68**	**0.70**		**0.71**	**0.73**	

Item 11	**0.62**	**0.63**	0.01	**0.52**	**0.53**		**0.49**	**0.50**	

Item 12	**0.49**	**0.55**	-0.06	**0.56**	**0.57**		**0.50**	**0.51**	

*Note.* Loadings > .3 are in bold. EFA = Exploratory Factor Analysis. CFA = Confirmatory Factor Analysis

**Confirmatory factor analysis (Samples 2 and 3).** In both samples, we evaluated a one- and two-factor model. Fit indices and factor loadings for Sample 2 and Sample 3 are presented in Table 2. A Chi-Square difference test to compare the two models could not be calculated given that the models were not nested (item 1 was dropped in the two-factor model), so we interpreted the models based on fit indices and the patterns of factor loadings. *CFI* and *TLI* approached the 0.95 cutoff for both models in both samples (range 0.89–0.94). *RMSEA* indicated an acceptable fit (0.06) for a one- and two-factor model in Sample 2, and was slightly above cut-of for both models in Sample 3 (0.07). The Chi-square tests were significant. However, this is most likely because of the large sample. For both samples, factor loadings in the one-factor model were above .30 except for item 1 and item 6. Corrected item-total correlations were calculated and are presented in Tables 3, 4, and 5. Consistent with the factor loadings, items one and six have low correlations with the corrected total score (< .30) in at least two of the samples. Item-total correlation for item four was also below .30 in two samples.

**Table 3:** Corrected item-total correlations and inter-item correlation matrix of Sample 1

	Corrected item-total correlation	1	2	3	4	5	6	7	8	9	10	11	12

Item 1	.26	-											

Item 2	.39	.14	-										

Item 3	.38	.18	.21	-									

Item 4	.28	.20	.07	.20	-								

Item 5	.43	.19	.25	.26	.14	-							

Item 6	.33	.06	.35	.13	.13	.22	-						

Item 7	.36	.13	.23	.26	.20	.23	.16	-					

Item 8	.43	.24	.11	.47	.32	.28	.12	.24	-				

Item 9	.54	.22	.33	.31	.21	.35	.29	.29	.44	-			

Item 10	.56	.17	.42	.35	.14	.49	.31	.29	.35	.49	-		

Item 11	.49	.14	.36	.24	.20	.25	.32	.24	.31	.43	.48	-	

Item 12	.39	.11	.32	.21	.16	.31	.20	.19	.17	.30	.34	.39	-

*Note.* Corrected item-total correlations are *Pearson* correlations coefficients. Inter-item correlations are polychoric.

**Table 4:** Corrected item-total correlations and inter-item correlation matrix of Sample 2

	Corrected item-total correlation	1	2	3	4	5	6	7	8	9	10	11	12

Item 1	.20	-											

Item 2	.36	.17	-										

Item 3	.37	.05	.22	-									

Item 4	.28	.12	.15	.19	-								

Item 5	.39	.11	.23	.22	.23	-							

Item 6	.15	.05	.24	.08	.13	.09	-						

Item 7	.30	.06	.05	.20	.15	.22	.06	-					

Item 8	.44	.18	.25	.45	.14	.20	.08	.26	-				

Item 9	.44	.16	.18	.26	.17	.27	.13	.24	.37	-			

Item 10	.52	.19	.41	.22	.17	.41	.09	.25	.24	.41	-		

Item 11	.41	.11	.23	.19	.24	.20	.07	.26	.26	.19	.39	-	

Item 12	.43	.13	.27	.27	.19	.22	.00	.21	.26	.31	.39	.40	-

*Note.* Corrected item-total correlations are *Pearson* correlations coefficients. Inter-item correlations are polychoric.

**Table 5:** Corrected item-total correlations and inter-item correlation matrix of Sample 3

	Corrected item-total correlation	1	2	3	4	5	6	7	8	9	10	11	12

Item 1	.15	-											

Item 2	.31	.16	-										

Item 3	.37	.16	.27	-									

Item 4	.32	.13	.07	.28	-								

Item 5	.47	.11	.28	.22	.22	-							

Item 6	.21	.01	.12	.07	.22	.19	-						

Item 7	.38	.09	.15	.24	.30	.19	.18	-					

Item 8	.32	.04	.03	.37	.23	.18	.08	.36	-				

Item 9	.49	.04	.23	.25	.24	.39	.21	.35	.42	-			

Item 10	.52	.07	.35	.34	.19	.53	.26	.30	.24	.47	-		

Item 11	.38	.14	.29	.18	.13	.35	.08	.16	.09	.30	.39	-	

Item 12	.40	.07	.22	.32	.24	.34	.03	.26	.18	.31	.28	.35	-

*Note.* Corrected item-total correlations are *Pearson* correlations coefficients. Inter-item correlations are polychoric.

The two-factor model had, in both samples, loadings above .30 for items in each factor. However, again, item 6 did not reach .30. Factor 1 and Factor 2 were highly correlated in the two-factor model (.77 in Sample 2 and .75 in Sample 3).

We accepted the parsimonious one-factor model as final for the following reasons: Fit indices did not strongly differ between the two models, and in the two-factor model, the factors were highly correlated. Moreover, in the two-factor model, the second factor only contains three items that were not, in our view, coherent.

**Reliability.** Internal consistency was assessed by calculating Cronbach’s alpha in the three samples (Table 1) and was considered to be acceptable. Inter-item correlations for each sample are presented in Tables 3, 4, and 5. We used polychoric correlations because the items are ordinal in nature. Despite of an acceptable Cronbach’s alpha, inter-item correlations were medium to small. Weak inter-item correlations were especially observed for item one (Sample 1, 2, and 3), item four (Sample 2) and item six (Samples 2 and 3). These items were already considered to be suboptimal in the corrected item-total correlations. Five months after the mass-testing session of Sample 3, the SUIS was re-administered in an independent experimental study with 52 students – 49 of these students could be matched with Sample 3, resulting in an intraclass correlation coefficient (*ICC*, McGraw & Wong, 1996) of .69, *N* = 49, assuming absolute agreement and a mixed model using single measures.

**Convergent validity.** Total SUIS score was, as predicted, inversely correlated with the sum score of the VVIQ, *r*(350) = -.35, *p* < .001 as well as with the visual subscale of the QMI, *r*(338) = -.38, *p* < .001.

## Discussion

We created a Dutch version of the Spontaneous Use of Imagery Scale and evaluated its psychometric properties. First, we examined the underlying factor structure of the SUIS. Exploratory factor analysis was followed by confirmatory factor analysis using independent samples. Second, we examined reliability and convergent validity. A parsimonious one-factor model was preferred above the two-factor model, suggesting that there is one underlying component: general use of visual mental imagery.

Evidence for a one-factor model was found in students, but also in a heterogeneous sample with broader age range and educational background. We investigated two formats of the SUIS: pencil-and-paper and online administration. Three items were suboptimal, suggesting that they could be revised or dropped. If a short version of the SUIS were desired, items one, four, and six might be excluded with Dutch-speaking populations. Based on confirmatory factor analysis and classical test theory analyses, they do not adequately measure the general imagery factor. Our reading of the items indicates that these three items are less straightforward and are not restricted to “making images in a certain situation”.

It is likely that other variables, other than use of imagery, influence SUIS scores. For example, some people do not read novels (item four) or do not read information about technical material (item six). Thus, these items would be infrequently endorsed, regardless of the propensity to use imagery. The use of a GPS or complex mobile phone, for example, might influence the score on item one.

With regard to reliability, Cronbach’s alpha was similar in the three datasets and within an acceptable range. Additionally, test-retest reliability, evaluated by a subsample, was adequate. It is unclear, however, why the corrected item-total correlations were remarkably lower in the Dutch compared to the English version.

As predicted, the SUIS total score was related with higher levels of visual imagery ability (VVIQ and QMI), comparable to Reisberg et al. (2003) who found that a subgroup of participants with high vividness ability scored higher on the SUIS compared to a ‘low-vividness’ subgroup. The medium effect sizes of the correlations suggest that VVIQ, QMI and SUIS are related, although not interchangeable, constructs. The VVIQ and QMI primarily focus on the vividness and quality of mental imagery (see Materials). The SUIS asks how likely it is that people will have or will use mental images in certain situations. Image quality or vividness is only mentioned in item 5 of the SUIS. Moreover, in the VVIQ and QMI participants have to rate the image in the moment, whereas the SUIS concerns more general statements.

Our three samples indicated that the vast majority of people experience visual imagery in daily life. All participants experienced imagery in at least one of the 12 situations, but 2.3% of the participants reported no imagery in more than half of the situations. Mean total scores were in line with previous research. In 15 studies reporting mean SUIS scores in non-clinical populations, including the original study of Reisberg et al. (2003), the total mean score ranged between 36.4 and 40.8, with standard deviations ranging from 6.2 to 9.3 (Berna, Lang, Goodwin, & Holmes, 2011; Davies et al., 2012; Deeprose & Holmes, 2010; Deeprose, Malik, & Holmes, 2012; Holmes, Coughtrey, & Connor, 2008; Holmes, Lang, et al., 2008; Holmes, Mathews, Mackintosh, & Dalgleish, 2008; Krans, Näring, Holmes, & Becker, 2010; Krans, Näring, Speckens, & Becker, 2011; Lang, Moulds, Holmes, 2009; Mast et al., 2003; McCarthy-Jones et al., 2012; Murphy, Barnard, Terry, Cathery-Goulart, & Holmes, 2011; Nelis, Vanbrabant, Holmes, & Raes, 2012). Two experimental studies (Holmes, Lang, & Shah, 2009) reported notable higher mean scores in their conditions (mean item scores ranging from 3.7 to 4.0, which are equivalent with a total score ranging from 43.9 to 48.0). We found no significant relation between imagery use and age. Concerning gender difference, females reported to make significantly more use of mental imagery compared to men. This result parallels some previous findings showing that females have more vivid images than males (e.g., in first-year psychology students; White, Ashton, & Brown, 1977).

Our paper has several implications. With regard to the scoring of the questionnaire, the use of a total score is recommended given the unidimensional underlying structure. Given the large sample, the means and standard deviations could be used as norms for future studies. Further research is needed to investigate psychometric properties across different groups, however. Corrected item-total correlations were much lower than reported for the English version in Reisberg et al. (2003). Future research could address this point by investigating the questionnaire across multiple languages. It would also be interesting to have a concurrent imagery task to evaluate the relation between the self-report measure and a performance measure (Pearson et al., 2013) and also to further examine the ecological validity. This may involve not only questionnaires, but also think-aloud tasks to assess spontaneous use of imagery during a range of daily tasks. There is still room for improvement of the Dutch SUIS, therefore future research can also further investigate and improve the psychometric properties of the SUIS. This might be achieved by adding additional items or revising existing items. Moreover, the SUIS can be used in psychopathology research to determine how imagery is involved in various psychiatric disorders. Finally, the SUIS could be useful to link individual’s use of imagery to other aspects of cognition (e.g., the vividness of memories).

The present research has limitations. First, the online selection of respondents from the community creates a heterogeneous sample, but it limits control over the environment conditions and standardization of the testing environment. Second, in all samples, there were more women than men. Given that we found differences between men and women, it would be interesting to have larger subsamples of men.

In summary, the present studies offer the first support for a one-factor structure of (the Dutch version of) the SUIS, an instrument that is being widely used to assess general use of mental imagery in daily life in both non-clinical and clinical samples. Reliability of the questionnaire was acceptable and the SUIS had convergent validity, expressed in associations with other imagery questionnaires.

## Author Information

Sabine Nelis is a research assistant of the Research Foundation – Flanders (Fonds Wetenschappelijk Onderzoek Vlaanderen). James W. Griffith is supported by the Research Foundation-Flanders (FWO; GP.035.11N; G.0339.08). Emily Holmes is supported by the Medical Research Council (United Kingdom) intramural program [MC-A060-5PR50], the Lupina Foundation, a Wellcome Trust Clinical Fellowship [WT088217], and the National Institute for Health Research (NIHR) Oxford Biomedical Research Centre based at Oxford University Hospitals NHS Trust, Oxford University [REF A93182]. The views expressed are those of the author(s) and not necessarily those of the NHS, the NIHR or the Department of Health.

## Appendix

Reisberg, D., Pearson, D.G., & Kosslyn, S.M. (2003). Intuitions and Introspections about Imagery: The Role of Imagery Experience in Shaping an Investigator’s Theoretical Views. *Applied Cognitive Psychology, 17*, 147–160.

Lees de volgende beschrijvingen en duid aan in welke mate elke beschrijving op jou van toepassing is. Denk niet te lang na over elke beschrijving, maar antwoord op basis van jouw gedachten over hoe je de activiteit wel of niet zou uitvoeren.

Indien een beschrijving altijd volledig van toepassing is, schrijf dan “5”,

Indien het nooit van toepassing is, schrijf dan “1”,

Indien de beschrijving voor de helft van de tijd van toepassing is, schrijf dan “3”,

En gebruik de andere nummers (2 en 4) overeenkomstig.

_____ 1. Wanneer ik naar een nieuwe plaats ga, heb ik het liefst aanwijzingen die gedetailleerde beschrijvingen bevatten van oriëntatiepunten (zoals de grootte, vorm en kleur van een tankstation) naast de namen van die oriëntatiepunten.
_____ 2. Wanneer ik een blik opvang van een auto die deels verborgen is achter struiken, dan “vervolledig” ik de auto automatisch door de auto in zijn geheel visueel voor te stellen in mijn hoofd.
_____ 3. Wanneer ik in een winkel op zoek ben naar nieuwe meubels, maak ik mij altijd een voorstelling van hoe de meubels er zouden uitzien op bepaalde plaatsen in mijn huis.
_____ 4. Ik verkies om romans te lezen die me er gemakkelijk toe brengen om voor te stellen waar de personages zijn en wat ze aan het doen zijn, in plaats van romans die moeilijk visueel voor te stellen zijn.
_____ 5. Wanneer ik er aan denk een familielid te bezoeken, heb ik bijna altijd een duidelijk mentaal beeld van hem of haar.
_____ 6. Wanneer relatief gemakkelijk technisch materiaal duidelijk beschreven wordt in een tekst, vind ik illustraties afleidend omdat ze interfereren met mijn bekwaamheid om het materiaal visueel voor te stellen.
_____ 7. Als iemand me zou vragen om getallen die uit twee cijfers bestaan op te tellen (bv. 24 en 31), dan zou ik ze visueel voorstellen, wat me helpt om de getallen daarna op te tellen.
_____ 8. Voor ik mij aankleed om uit te gaan, stel ik mij eerst voor hoe ik er zal uitzien als ik de verschillende klerencombinaties draag.
_____ 9. Wanneer ik denk over een reeks boodschappen die ik moet doen, stel ik mij de winkels die ik ga bezoeken voor.
_____ 10. Wanneer ik eerst de stem van een vriend of vriendin hoor, komt er bijna altijd een visueel beeld van hem of haar in mijn hoofd op.
_____ 11. Wanneer ik een radio-omroeper of een DJ hoor die ik nog nooit in het echt heb gezien, dan stel ik mezelf gewoonlijk voor hoe die er zou uitzien.
_____ 12. Wanneer ik een auto-ongeluk zou zien, zou ik mij een voorstelling maken van wat er gebeurd is wanneer ik later de details probeer te herinneren.
